# 1478. Evaluation Of Anal Cancer Screening Program in men who have sex with men (MSM) persons with HIV (PWH) At Two academic center HIV Clinics 2018-2022

**DOI:** 10.1093/ofid/ofad500.1314

**Published:** 2023-11-27

**Authors:** Amit Achhra, Elizabeth Chan, Serina Applebaum, Maggie Guerrero, Ritche Hao, Haddon Pantel, Michael D Virata, Lydia A Barakat

**Affiliations:** Yale school of Medicine, New Haven, Connecticut; Yale school of Medicine, New Haven, Connecticut; Yale School of Medicine, New Haven, Connecticut; Yale school of Medicine, New Haven, Connecticut; Yale school of Medicine, New Haven, Connecticut; Yale school of Medicine, New Haven, Connecticut; Yale University School of Medicine, New Haven, Connecticut; Yale School of Medicine, New Haven, Connecticut

## Abstract

**Background:**

Treatment of anal dysplasia has been shown to reduce incidence of squamous cell carcinoma of the anus (SCCA) in PWH, particularly in MSM. Annual anal cytology (PAP) has been offered for SCCA screening since ∼2010 at our urban academic center ambulatory HIV clinics, and those with abnormal PAP (atypical squamous cells of undetermined significance (ASCUS), or Anal Intraepithelial Neoplasia (AIN) 1-3) are referred for high-resolution anoscopy (HRA) and dysplasia treatment. Evaluation of screening cascade can help better understand and improve SCCA screening protocols.

**Methods:**

We performed a retrospective chart review (2018-2022) on MSM PWH, age 35 and older, enrolled at our two HIV clinics who had at least one clinic visit by 12/31/2019. Of total eligible for SCCA screening, we calculated proportion of those engaged (received at least one screening (PAP or HRA)) and retained (received at least one subsequent screening) in care. Characteristics of people who did not engage in screening were evaluated by logistic regression.

**Results:**

A total of 432 individuals were eligible for SCCA screening. The median [interquartile range] age was 57 [48-62] years, 97% were on antiretroviral therapy, 28% were Blacks, 62% had history of smoking, and 24% had a prior history of anal dysplasia. A total of 219 (50.7%) engaged in screening, and only 113 (26%) were retained (Figure-1). Seventy-four individuals had at least one abnormal PAP during follow-up, of which 35 (47%) received HRA at least once. In multivariable analysis, older age and history of smoking negatively correlated with engagement while history of anal dysplasia positively correlated with engagement (table-1). Race (Blacks vs other) and type of insurance (public/private) did not correlate with engagement.

**Figure:**
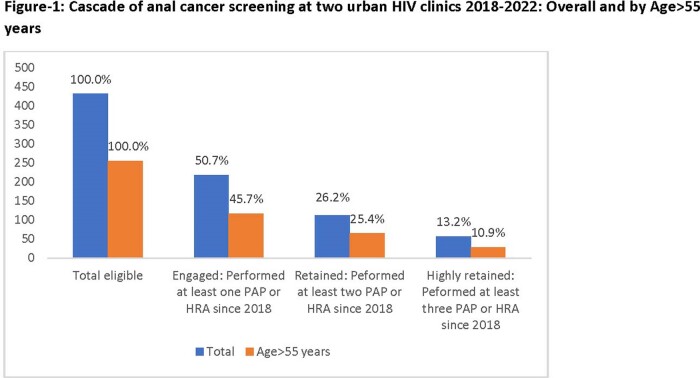

**Figure:**
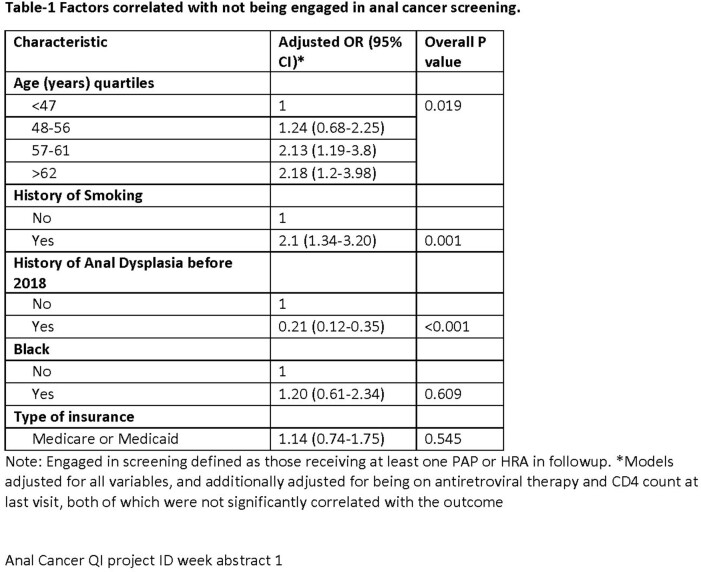

**Conclusion:**

We noted high rates of loss of engagement and retention in SCCA screening even in PWH highly engaged in HIV care. Older MSM PWH, who are at higher risk of SCCA, were less likely to be engaged in screening.

**Disclosures:**

**Michael D. Virata, MD, FACP**, Gilead Sciences: Advisor/Consultant|Janssen: Advisor/Consultant|ViiV Healthcare: Advisor/Consultant

